# Dr. Samuel W. Wetmore

**Published:** 1906-07

**Authors:** 


Dr. Samuel W. Wetmore, of Buffalo, (U. of B. '62) died at his home May 28, 1906, aged 74 years. He had been in poor health for some years but his fatal illness,—cerebral hemorrhage,—was of comparatively short duration. He was for a short time demonstrator of anatomy at the University of Buffalo and he also served as contract surgeon at Fort Porter during a portion of the Civil War. He was professor of surgery in the "Buffalo college of rational Medicine," during its existence and was at one time health physician of Buffalo. About twenty years ago Dr. Wetmore married Dr. Mary Berkes, who is also a graduate of the University of Buffalo, and who survives.

